# Accelerating bioinformatics implementation in public health

**DOI:** 10.1099/mgen.0.001051

**Published:** 2023-07-10

**Authors:** Kevin G. Libuit, Emma L. Doughty, James R. Otieno, Frank Ambrosio, Curtis J. Kapsak, Emily A. Smith, Sage M. Wright, Michelle R. Scribner, Robert A. Petit III, Catarina Inês Mendes, Marcela Huergo, Gregory Legacki, Christine Loreth, Daniel J. Park, Joel R. Sevinsky

**Affiliations:** ^1^​ Theiagen Genomics, Suite 400, 1745 Shea Center Drive, Highlands Ranch, CO, 80129, USA; ^2^​ Wyoming Public Health Laboratory, 208 S College Dr, Cheyenne, WY 82007, USA; ^3^​ Broad Institute of Harvard and MIT, 415 Main St, Cambridge, MA 02142, USA

**Keywords:** bioinformatics, epidemiology, genome, public health, sequencing, Terra

## Abstract

We have adopted an open bioinformatics ecosystem to address the challenges of bioinformatics implementation in public health laboratories (PHLs). Bioinformatics implementation for public health requires practitioners to undertake standardized bioinformatic analyses and generate reproducible, validated and auditable results. It is essential that data storage and analysis are scalable, portable and secure, and that implementation of bioinformatics fits within the operational constraints of the laboratory. We address these requirements using Terra, a web-based data analysis platform with a graphical user interface connecting users to bioinformatics analyses without the use of code. We have developed bioinformatics workflows for use with Terra that specifically meet the needs of public health practitioners. These Theiagen workflows perform genome assembly, quality control, and characterization, as well as construction of phylogeny for insights into genomic epidemiology. Additonally, these workflows use open-source containerized software and the WDL workflow language to ensure standardization and interoperability with other bioinformatics solutions, whilst being adaptable by the user. They are all open source and publicly available in Dockstore with the version-controlled code available in public GitHub repositories. They have been written to generate outputs in standardized file formats to allow for further downstream analysis and visualization with separate genomic epidemiology software. Testament to this solution meeting the requirements for bioinformatic implementation in public health, Theiagen workflows have collectively been used for over 5 million sample analyses in the last 2 years by over 90 public health laboratories in at least 40 different countries. Continued adoption of technological innovations and development of further workflows will ensure that this ecosystem continues to benefit PHLs.

## Data Summary

All Theiagen workflows are available on GitHub (https://github.com/theiagen) in tagged version releases. All workflows can be imported into analytical platforms, including any Terra workspace, from Dockstore (https://dockstore.org/organizations/Theiagen).

Sequence data used in this publication are available in the National Center for Biotechnology Information (NCBI) Sequence Read Archive under the accession numbers listed in Table S1, available in the online version of this article. All analyses have been undertaken via the Terra platform in a publicly available workspace, which includes the sequence data, relevant workflows imported into the workspace and workflow results (https://app.terra.bio/#workspaces/theiagen-demos/MGen-Theiagen-2023).

**Impact Statement**

We describe the infrastructure used to successfully implement bioinformatic analyses of microbial genomes in public health laboratories using the Terra platform and Theiagen Genomics (Theiagen) bioinformatics workflows. This open-source infrastructure can be adopted at low cost by public health practitioners without a coding skill set, and achieve reproducible, validated results from standardized analyses. The use of a web-based interface and the cloud for data analysis and storage enables users with an internet connection to undertake analyses at a scale that suits their needs, analysing between a handful to hundreds of thousands of genomes from anywhere in the world. Our open-source, containerized workflows have been tailored for public health purposes. They are available for importing sequence data into the Terra workspace from sequencers and public repositories; genome assembly, quality control and characterization; phylogenetic analyses; and data sharing. Use of this infrastructure is demonstrated in a case study data analysis using previously published genomes. This recapitulates previously published results and, importantly, illustrates the results that can be obtained with this infrastructure. This ecosystem has been applied widely in many PHLs and could be adopted in many more.

## Introduction

Microbial genomics is becoming an increasingly critical function for public health laboratories (PHLs) [1]. Sequence data are used by PHLs for the identification and characterization of pathogens; surveillance of population structure and key features of interest, such as antimicrobial resistance determinants; and assessment of transmission, including outbreak investigations. While incredible progress has been made towards the routine generation of microbial sequencing data for public health [2, 3], bioinformatic analysis of these data remains a major challenge in many PHLs [4, 5 ].

Bioinformatics software has been developed to address many of the most pertinent questions for public health practitioners but the implementation of these bioinformatics tools in PHLs is challenging in practice. Implementation requires adherence to the key principles of public health analyses: standardization of methods, reproducibility of results, and thorough validation and auditability. Additionally, bioinformatics solutions for PHLs must be scalable, portable and secure for any amount of data analysis anywhere in the world. They must be consistently accessible (open-source), low cost, and easy to use without the need for code. To achieve these requirements for public health bioinformatics, we share the views of Black *et. al* [4] regarding their recommendations for an ‘open bioinformatics ecosystem’ that users can access via a web-based graphical user interface (GUI). This should consist of a registry of versioned and validated containerized workflows for genomic analyses, written in a standard workflow language, to produce reproducible, auditable results, while leveraging cloud computing where available. Subsequent integration with sensitive epidemiological metadata (e.g. personally identifiable information) and downstream analyses and visualization could occur on the user’s local computer system or in the cloud environment, depending on local regulations about how these data should be used.

Technological innovations across the field of microbial genomics have provided the foundation for the open bioinformatics ecosystem needed in public health. Containerization of microbial genomics software has facilitated the use of standardized software within standardized environments, despite varied computing ecosystems. This has greatly increased the reproducibility of analyses whilst making open-source software easier to share and use [6, 7]. The State Public Health Bioinformatics (StaPH-B) docker-builds project was created by a community of public health bioinformaticians to containerize software for public health applications. As of February 2023, StaPH-B hosts 142 containerized images of microbial genomics software – some of which have over 1 million downloads [8–10]. The use of workflow management systems has enabled the standardization of modular analytical ‘workflows’ by specifying and executing the flow of data through a series of processes using software containers in a stepwise manner [11, 12]. Frequently used workflow systems include the NextFlow language and execution engine [13]; the Cromwell execution engine using Workflow Description Language (WDL) [14] and Common Workflow Language (CWL) [15] and SnakeMake [16], which each have multiple execution engines. Open-source repositories, such as GitHub and Dockstore, make version-controlled workflows in frequently used languages publicly available and discoverable. Workflow management systems also have built-in logic for scalable analysis across a variety of computing infrastructures. Since the cost of computing has decreased significantly, whether via local computers, high-performance clusters or cloud platforms, large-scale analyses are now more financially feasible for PHLs. These innovations standardize bioinformatics analyses and together enable the reproducible, scalable, accessible and portable bioinformatics that is needed in public health.

Several efforts have used these recent technological advancements to create workflows for microbial genome analysis. Notable open-source examples that address the requirements for public health bioinformatics include Bactopia [17], a flexible pipeline for performing comprehensive bacterial analysis; the viral-pipelines repository for the analysis of viral next-generation sequencing (NGS) data [18]; and the Augur toolkit for tracking evolution from sequence data [19]. To use these workflows, users typically need access to their own computing infrastructure and the ability to use code and a command line interface.

Efforts have been made to broaden access to bioinformatics beyond the technical community through the use of open-access GUI web applications with scalable back-end infrastructures [20–23]. These platforms have shown incredible promise to proliferate bioinformatic analyses, especially in PHLs that lack personnel with a coding skillset. One platform that is uniquely fitting for PHLs to adhere to the requirements for public health bioinformatics is Terra [24]. Terra has innate compatibility with open-source, containerized workflows; a scalable and secure cloud computing back-end; and a built-in auditing system. The platform itself is open-source, freely accessible, and jointly developed and supported by the Broad Institute (Cambridge, MA, USA), Microsoft Corporation (Redmond, CA, USA), and Verily Life Sciences (South San Francisco, CA, USA). It grants users access to a registry of over a two thousand version-controlled workflows and cloud computing resources through a web-based, click-button interface. Users can easily launch workflows, monitor job submissions, and organize results, allowing for the rapid adoption of reproducible and standardized bioinformatics workflows that can be validated internally and by users in adherence with individual quality management systems.

Here, we describe a method for bioinformatics implementation that takes advantage of these technological innovations to build a standardized, reproducible and easily accessible bioinformatics ecosystem for PHLs. This bioinformatics ecosystem is based on the development and distribution of open-source, containerized workflows using a standard workflow language, WDL, that are made accessible through the Terra platform. We discuss the Terra infrastructure, alignment with key requirements for public health bioinformatics, and ease of use for scalable analyses of pathogen sequence data. Additionally, we provide insight into how Terra has been successfully adopted by many PHLs across the world, all of which vary in levels of bioinformatics expertise and prioritization of different pathogens.

## Theory and implementation

### An open bioinformatics ecosystem for public health

**Fig. 1.**
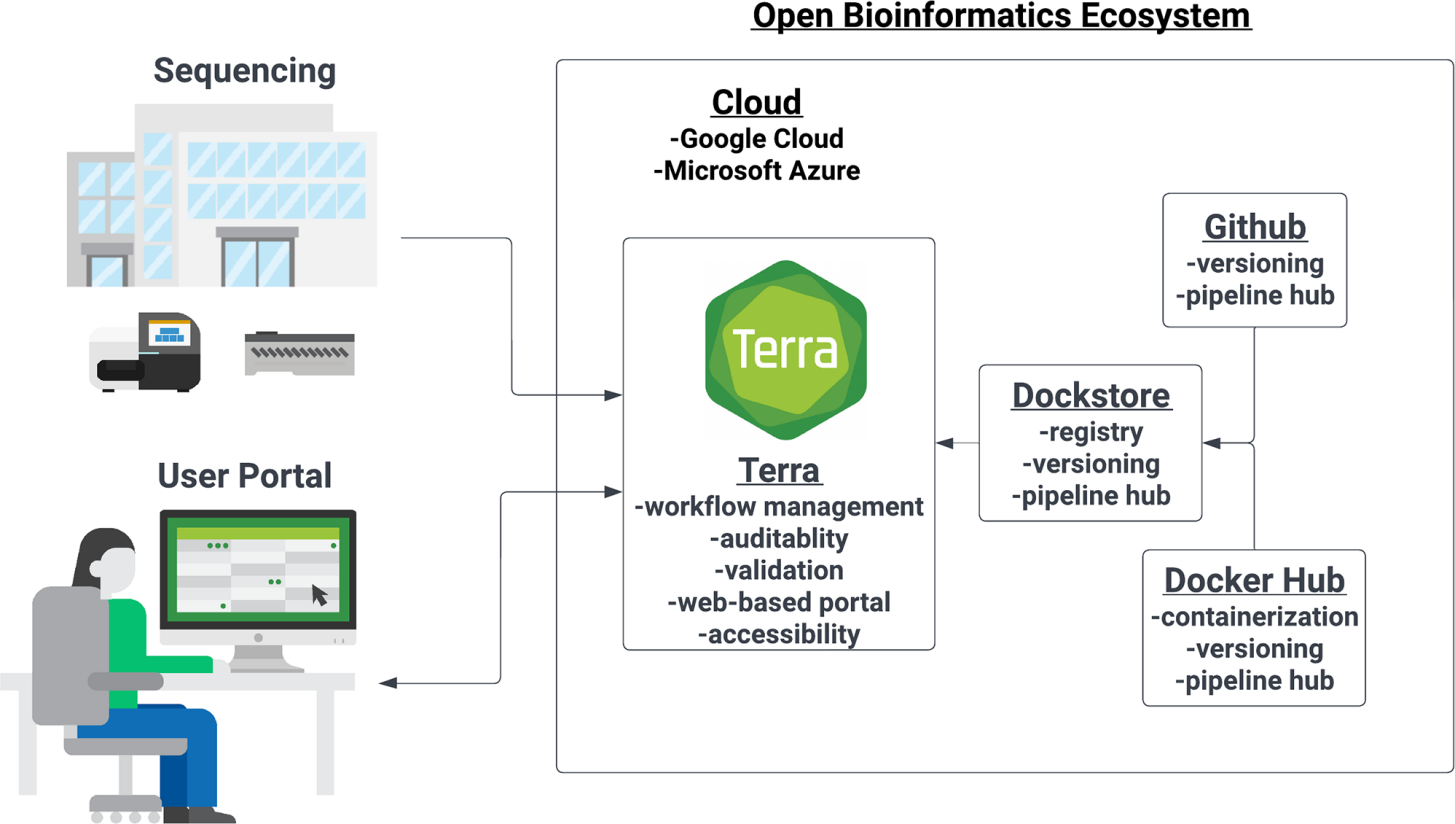
The open bioinformatics ecosystem. Our ecosystem centres around the use of Terra and the Cloud computing platforms. We have labelled the functions of the primary components of this ecosystem using the same vocabulary as Black *et al.* GitHub – a code hosting platform for version control and collaboration – hosts the code for WDL workflows and dockerfiles to build Docker images. Docker Hub is a hosted library of standardized, validated container images used by WDL workflows. Terra will retrieve versioned images from Docker Hub during WDL workflow execution. Dockstore is an open registry for sharing interoperable tools and workflows. Terra pulls versioned workflows directly from Dockstore into Terra workspaces. Terra is a workflow orchestration platform that enables workflow management, auditability and validation using sharable secure workspaces. Terra also provides a browser-based portal for easy accessibility and GUI functionality for non-bioinformatics scientists. Terra is hosted on the Google Cloud platform and Microsoft Azure. These provide a secure, scalable, distributable and inexpensive computing resource.

Terra was selected due to its adherence to the requirements for public health bioinformatics we previously defined for a bioinformatics ecosystem (Fig. 1). Bioinformatics workflows and cloud computing resources are made available to users through user-owned and controlled Terra workspaces. Each workspace consists of data tables for organized storage of sequence data and workflow outputs, and separate tabs for running workflows and assessing job history. Bioinformatics workflows are available to users through Dockstore, a platform for sharing reproducible software in adherence to GA4GH standards [25] and APIs [26]. Data storage and analysis on Terra are provided through the user’s cloud computing resources from either the Google Cloud Platform (GCP) or Microsoft Azure, in the geographical location specified by the user. Both Terra and its integrated cloud-computing platforms are private and secure, allowing access only to users individually authorized by the account owner and adhering to best practices in information security [27].

We have written 57 workflows to capture best practices for bioinformatic analysis of pathogen genomes and their associated metadata (Table S2, available in the online version of this article) that can be run through Terra. These Theiagen workflows have been developed alongside public health laboratories to tailor their functionality to public health needs. These have been written in WDL, a standard workflow language, and use containerized open-source software hosted on publicly accessible registries such as Docker Hub (https://hub.docker.com/) and Quay (https://quay.io/) (Table S3) [28–77]. We have containerized software as needed, adding this to the StaPH-B docker-builds project [6]. Workflows have been versioned and made publicly available on GitHub [78] and Dockstore [79]. The modular nature of the workflows allows for iterative development and improvements that are optimized for evolving public health applications in real time. Each version can be validated using cloneable public workspaces within Terra, where users can reproducibly run specified versions of pipelines on curated datasets (e.g. https://app.terra.bio/#workspaces/theiagen-validations/PHBG_Validation_v1-1-0). Automatic recording of the workflow information, including versions, parameters and inputs, ensures reproducibility of analyses and ease of auditing. Together, these features ensure that Theiagen workflows adhere to the requirements for public health bioinformatics we previously defined.

**Fig. 2.**
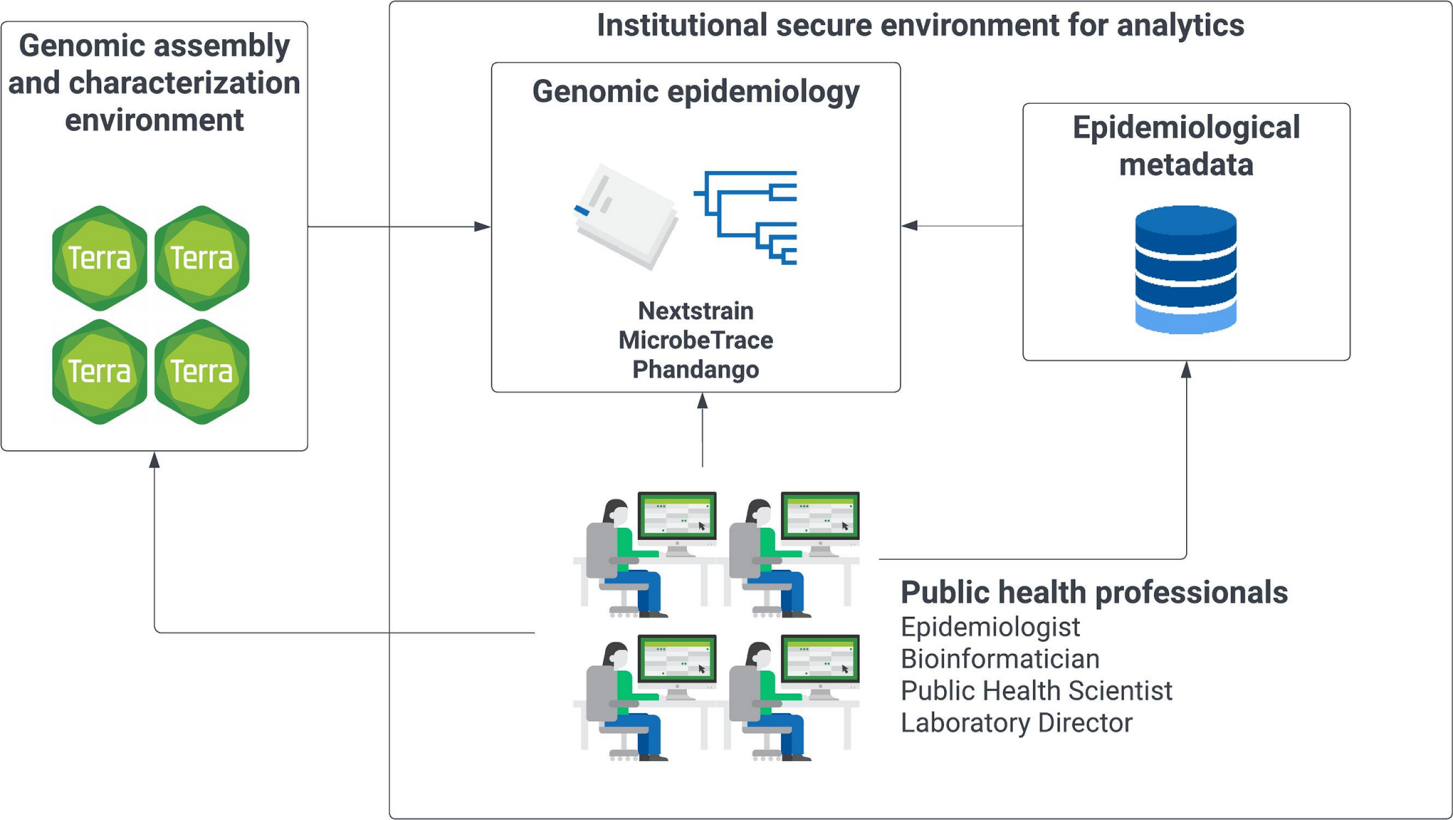
Separation of genomic assembly and characterization from analytics and genomic epidemiology. Workflow results and output files can readily be exported from the Terra ecosystem into institutionally controlled IT environments for integration with epidemiological metadata for further analysis and visualization.

Owing to the use of a workflow manager and containerized software, workflows can be used in a flexible manner. Users can execute a standardized workflow but update software containers and databases; substitute, add, or skip particular workflow modules; and modify parameters to best suit their needs. User-adjustable quality checks are implemented at multiple stages to ensure that outputs adhere to their individual quality management systems. Default quality metrics are specified by a consensus of international bioinformatics organizations, including the Centers for Disease Control and Prevention (CDC) PulseNet [80], Public Health Alliance for Genomic Epidemiology (PHA4GE) [81], CDC SARS-CoV-2 Sequencing for Public Health Emergency Response, Epidemiology and Surveillance (SPHERES) [82] and the StaPH-B community [83]. Outputs are provided in common non-proprietary file formats, e.g*.* fasta format for genome assemblies and Newick format for phylogenetic trees, which can be readily exported from the Terra platform. This facilitates integration with sensitive epidemiological metadata in a local computer environment for further analyses, visualization and interpretation of results (Fig. 2).

### Case study

We have undertaken example bioinformatics analyses via Terra with Theiagen workflows. Sequence reads representing bacterial isolates were selected from the Genomics and Food Safety (Gen-FS) *Salmonella enterica* ser. Bareilly whole-genome standards dataset [84, 85], Global Pneumococcal Sequencing (GPS) study dataset [National Center for Biotechnology Information (NCBI) BioProject PRJEB3084] [86] and CDC Division of Healthcare Quality Promotion (DHQP) dataset of OXA-48-like producing Carbapenem Resistant Organisms (NCBI BioProject PRJNA296771) [87]. Sequence Read Archive (SRA) accessions are provided in Table S1. Together, this set of Illumina paired-end reads represents multiple taxa of public health concern.

**Fig. 3.**
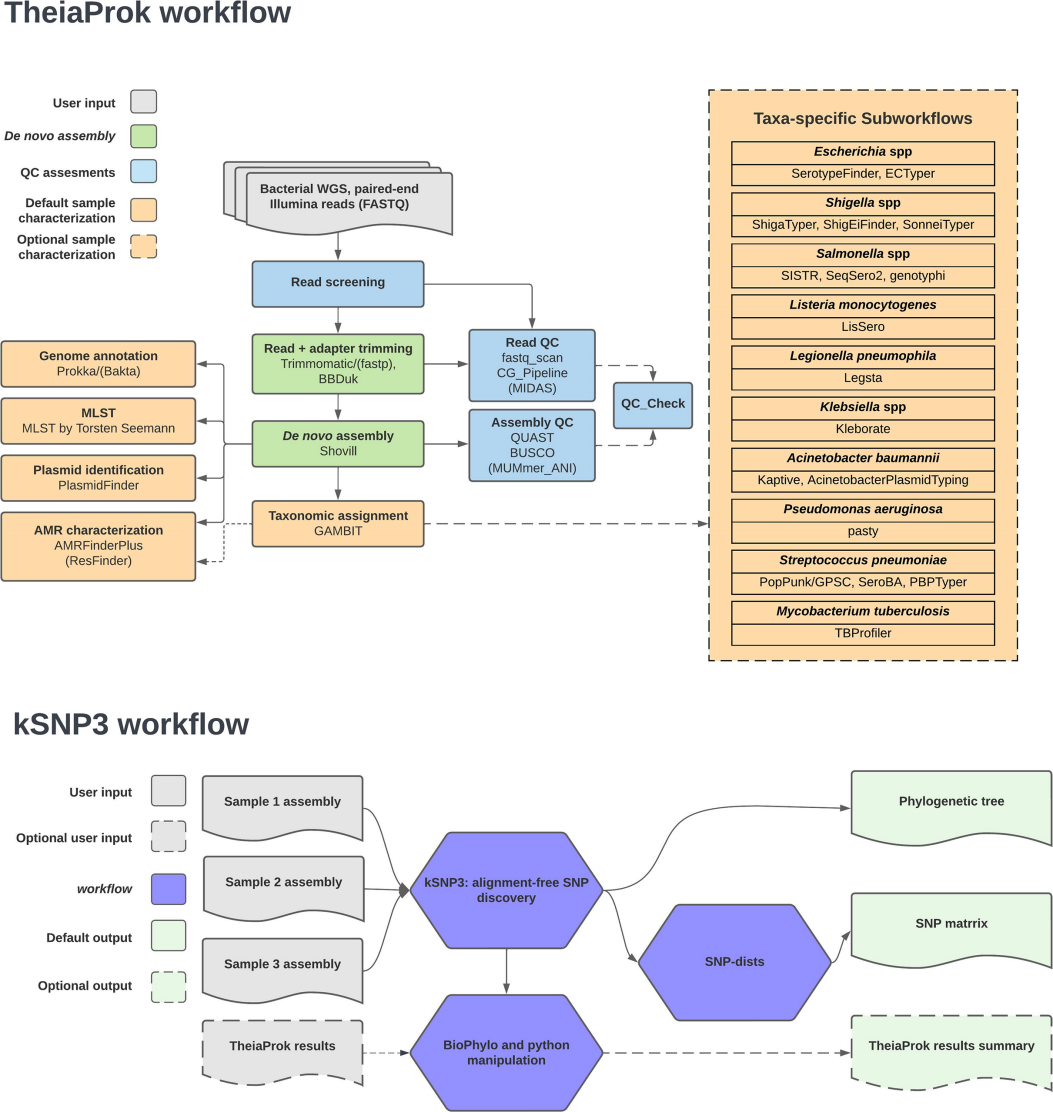
TheiaProk and kSNP3 workflow diagrams. Each workflow consists of inputs, processes and outputs represented by panels in the diagram. Dashed lines or parentheses indicate optional processes.

Sequence reads were brought into a Terra workspace using the SRA_Fetch v1.4.1 workflow. This workflow downloads read data from the NCBI’s SRA, requiring only the SRA or European Nucleotide Archive (ENA) run accession, and a user-defined sample name. All sequence data were analysed with the TheiaProk_Illumina_PE v1.1.1 workflow under default settings (Fig. 3). TheiaProk_Illumina_PE performs quality control, genome assembly, taxon assignment and genome characterization of Illumina paired-end reads from bacterial isolates. TheiaProk_Illumina_PE automatically activates taxon-specific analyses, e.g*. Salmonella* serotyping*,* based on *in silico* taxon identification. The Terra-compatible kSNP3 workflow [88] was used to generate a core-genome maximum-parsimony phylogeny, a pairwise SNP distance matrix and a summary of antibiotic-resistance genes (ARGs) and plasmids for the *S. enterica* ser. Bareilly samples. The resulting CSV files were exported from Terra, merged in Microsoft Excel and visualized in Phandango [89]. Instructions for running workflows in Terra are provided in Supplementary Material. The workspace where these analyses were performed has also been made publicly available: https://app.terra.bio/#workspaces/theiagen-demos/MGen-Theiagen-2023.

## Results

### Case study results

All sample sequence data were trimmed and assembled, and passed the pre-specified quality control thresholds described in Table S4. The characteristics of the samples, e.g. taxonomic identity and multilocus sequence type, and taxa-specific characteristics such as global pneumococcal sequence clusters for *S. pneumoniae,* were concordant with those that had been previously described (Tables S5–S7) [85–87]. The only discrepancies were between the antimicrobial resistance genes identified using AMRFinderPlus within the TheiaProk_Illumina_PE workflow, and those reported by NCBI Pathogen Detection for the CDC DHQP dataset of OXA-48-like producing Carbapenem Resistant Organisms. These discrepancies were primarily due to gene nomenclature differences and variations in databases between the tools. For example, for sample SRR3222446 the ‘blaOXA-9’ gene was reported by NCBI Pathogen Detection whereas TheiaProk reported a gene name of ‘blaOXA’ with ‘OXA-9 family’ specified only in the gene description (Table S7).

**Fig. 4.**
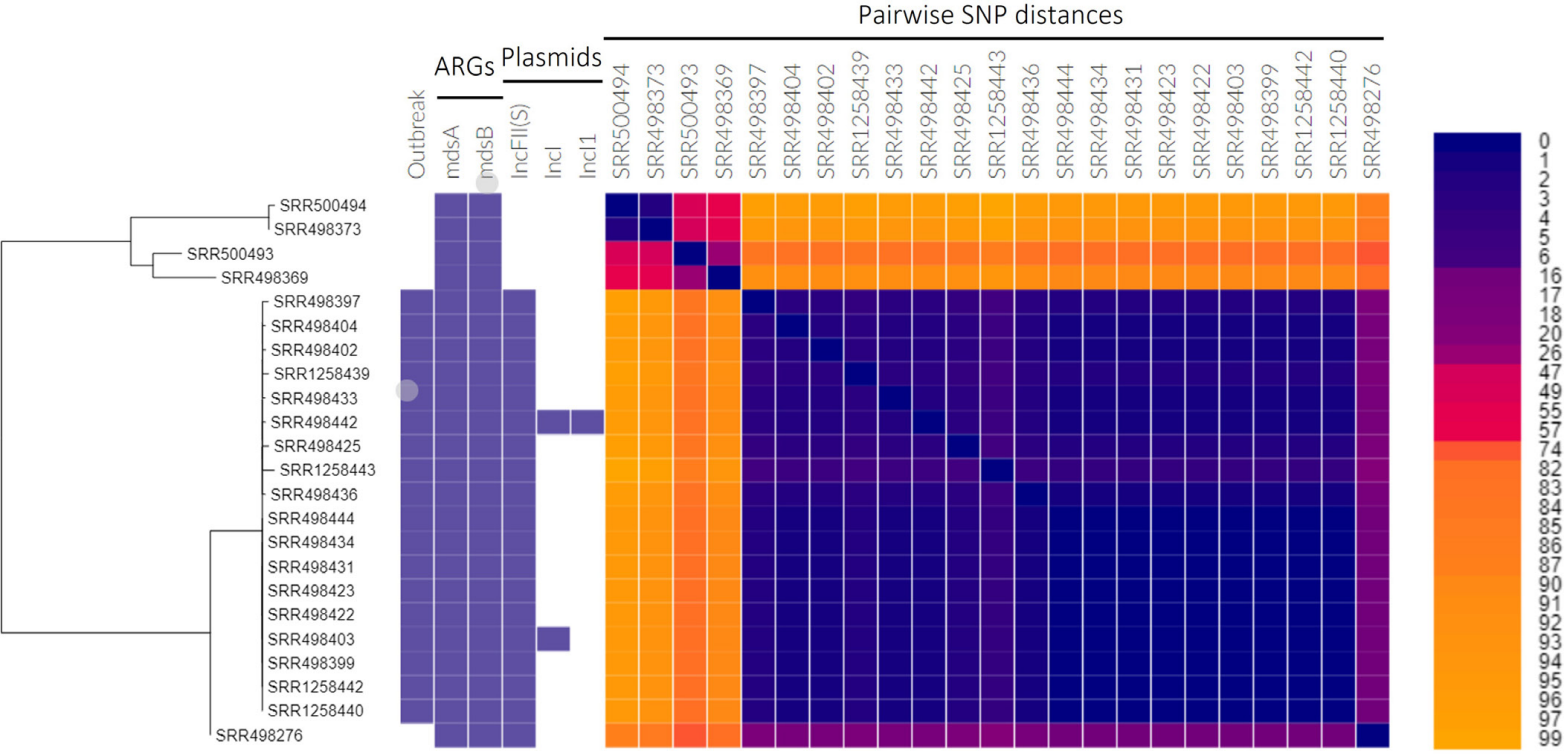
Core-genome phylogeny of *S. enterica* ser. Bareilly samples. Coloured boxes indicate that the genome was considered part of the outbreak in the original publication [85], the presence of given antibiotic resistance genes (ARGs) and plasmid types, and the number of pairwise SNPs between genomes. The phylogenetic tree in Newick format and CSV files containing the ARG and plasmid information were generated by the Theiagen kSNP3 workflow on Terra. The CSV files were merged in Microsoft Excel and then visualized in Phandango [89 ].

The *S. enterica* ser. Bareilly dataset is derived from a well-documented foodborne pathogen outbreak [84, 85]. Interactive visualization of phylogentic tree, antibiotic resistance gene, and plasmid summary output files in Phandango illustrated the relationship between genomes and facilitated interpretation of the epidemiology. The resulting phylogeny was confirmed to recapitulate the topology of the phylogenetic tree and the division of outbreak versus outgroup samples described in the publication for this dataset (Fig. 4) [85].

### Public health implementation

**Fig. 5.**
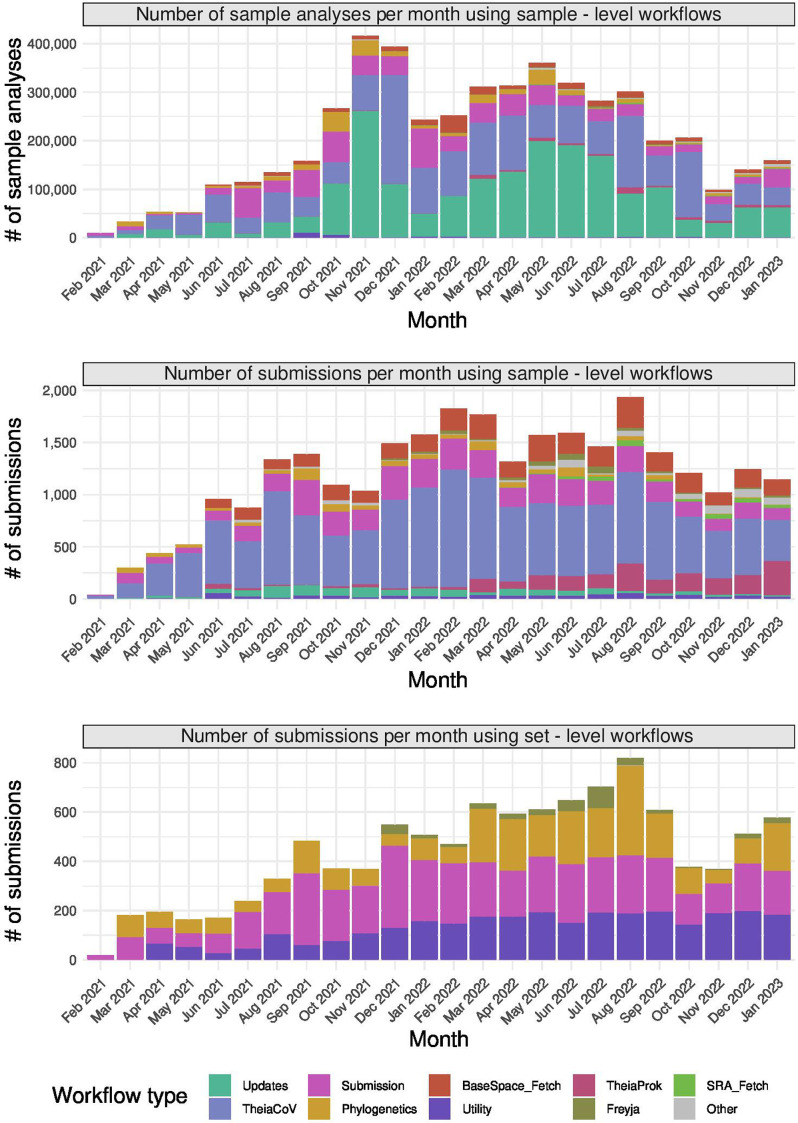
Number of sample analyses and workflows submitted using Theiagen workflows on Terra per month, from February 2021 to January 2023. The workflows have been grouped based on workflow name and/or functional category (Table S10). Sample-level workflows process individual samples, whereas set-level workflows process sets of samples, for example for comparative analyses. Users can launch multiple sample-level workflows simultaneously, creating a single submission for computation. For set-level workflows, a set of samples is processed in a single submission. Here, the top panel shows the number of sample analyses, each undertaken with a sample-level workflow. The central panel shows the number of submissions for sample-level workflows, which may launch analyses of multiple samples simultaneously. The bottom panel shows the number of submissions for set-level workflows, which is equivalent to the number of sets of samples analysed. The same sample may have been analysed multiple times. The raw data underlying these figures are presented in Tables S8–S10, with code to generate the analyses presented at https://github.com/theiagen/MGen-Theiagen-2023.

Within the Dockstore registry, Theiagen has published Terra-accessible, open-source, containerized workflows that are in active use in PHLs (Table S2). Using records of sample analyses collated by the Terra developers, we can see that Theiagen workflows have been used for 4926700 sample analyses and a total of 39111 workflow submissions from February 2021 to January 2023 (Fig. 5; Tables S8–S10). These data also show that many Theiagen workflows have been used by over 100 unique Terra users: the TheiaCoV_Illumina_PE workflow for viral genome analyses and TheiaProk_Illumina_PE workflow for bacterial genome analysis, for example, have been used by 188 and 126 unique Terra users, respectively, despite both of these workflows being released in the last year – a unique Terra user may also represent multiple personnel in the same laboratory. While exact numbers are unknown, these authors are aware of over 90 PHLs in more than 40 different countries that have accessed and used these workflows to analyse an estimated 900000 microbial genomes through the Terra portal. These public health laboratories are in low-, middle- and high-income countries in North America, Asia and Africa. Most operate on a national or state level, though some are focused on local public health jurisdictions or individual hospitals. Some have bioinformaticians undertaking command-line bioinformatics, though in many there is no bioinformatics expertise. These PHLs use Theiagen workflows in surveillance efforts, outbreak and cluster investigations, and for research purposes.

## Discussion

Implementation of bioinformatics in public health requires meeting the requirements of standardization of methods, reproducibility of results and thorough validation, with an open-source scalable, portable and secure infrastructure, at a low cost and with minimal training for non-expert users. The open bioinformatics ecosystem we have developed using Terra, Theiagen workflows, and separate visualization tools best meets these requirements.

In our open bioinformatics ecosystem, users can import their own and/or publicly available sequence data into private and secure Terra workspaces and choose from over a thousand version-controlled workflows hosted in the Dockstore repository for analysis. This includes open-source Theiagen workflows that have been developed specifically for public health applications to function akin to standard operating procedures (SOPs) used in the laboratory. As demonstrated in our case study, these standardized workflows empower scientists without coding skills to perform vital functions for public health genomics. Workflows are available to assemble, quality-control and characterize genomes of severe acute respiratory syndrome coronavirus 2 (SARS-CoV-2), influenza, human immunodeficiency virus (HIV), monkeypox virus, West Nile virus, or any bacterial or fungal pathogen, including enterics, healthcare-associated infections and multidrug-resistant organisms. Additional workflows are available for various phylogenetic analyses, data sharing with public repositories and efficient use of resulting data. Users can analyse any number of samples in a single workflow run owing to the scalable data storage and computing resources provided by the cloud, generating reproducible, validated and auditable results. Interpretable data are made available in common non-proprietary file formats that can be downloaded from Terra, integrated with metadata, and visualized and interpreted without the need for external data sharing.

The development of open-source, containerized workflows ensures the interoperability of our ecosystem’s constituent components across a multitude of bioinformatics infrastructures. Bioinformatics software has been containerized and made available on Docker Hub and Quay and used by thousands of scientists across a variety of computing environments [6, 7]. Hosting the workflows in GitHub and Dockstore enables importation and use of these workflows not only on Terra, but on other GUI platforms and through a command line interface as well (instructions to do so are provided in the Supplementary Information file). As files produced by the workflows are in common formats, visualization and interpretation of results can be achieved with many different tools. The modular and adaptable nature of the workflows means that they can be adopted for a diverse array of use cases, meeting the needs of individual PHLs and adhering to the FAIR (findable, accessible, interoperable and reusable) principles for research software, which should be adopted throughout the bioinformatics community [90].

A critical component of this bioinformatics ecosystem is the use of version control for both the software containers and workflows that utilize them. This facilitates iterative development of resources that are released under new versions, complementing our practitioner-led development approach whereby we develop functionality at the request of, and with, PHLs. Users provide real-time feedback during the development cycle, honing the function of these workflows to their needs. Frequent version releases provide users with analytical capabilities as soon as they are available, whilst maintaining stable code that contributes to the standardization of analyses and reproducibility of results. This reproducibility facilitates validation by individual PHLs, which will vary according to intended use and potential requirements imposed by the regulatory authorities governing their institution. This rapid inclusion of new features and analytical capabilities has provided the adaptable ecosystem needed for rapid response to emerging pathogens that was critical at the beginning of the SARS-CoV-2 pandemic and with the global emergence of the monkeypox virus [91].

To date, this open bioinformatics ecosystem using the Terra platform has been adopted by PHLs owing to its feasibility with the workforce and the financial resources available to laboratories. It is easily accessible to public health practitioners after minimal training, given Terra’s graphical user interface and the clear format of bioinformatic results. Training typically consists of instruction on how to use Terra and the workflows, and how to interpret results as part of epidemiological analyses. This ecosystem facilitates PHLs to undertake robust bioinformatic analyses without the need for a highly specialized workforce. Typically, this requires few personnel, as workflows can be used to analyse many samples in a single submission. For users of this ecosystem, cloud computing has been a better fit financially than on-premises computing solutions, given the elimination of capital expenditures and the ‘consumable’ nature of cloud computing. Resources can be budgeted for on a per-specimen basis, as they are for laboratory sequencing. This reduction in capital expenditures has been particularly beneficial in financially constrained settings, especially with the seamless scalability of cloud computing to grow (or shrink) with sequencing throughput. Testament to the feasibility of this ecosystem for implementing bioinformatics, millions of genome analyses have been performed on Terra using Theiagen workflows, not just limited to SARS-CoV-2, but many other pathogens of public health importance (Fig. 5).

Further development of the bioinformatics ecosystem will be facilitated by the open source and standardized approaches taken to build it. This might include the adoption of the NextFlow workflow management system, which is popular amongst bioinformaticians using a command line interface. Use of this infrastructure with alternative data storage and computation engines, such as additional cloud providers and on-premises solutions, will facilitate its use with a wider array of PHLs. Regardless of how bioinformatics technology develops, this ecosystem can be adapted to the evolving best practices and preferences of the community.

In conclusion, the open bioinformatics ecosystem offered by the Terra platform, Theiagen workflows and local data visualization solutions bridges the gap between the technical microbial bioinformatics community and the front-line public health workforce. The Theiagen workflows have been widely adopted for this purpose, and future developments will continue to embrace the best practices and opportunities provided by new technologies to continue to enhance bioinformatics implementation in public health.

## Supplementary Data

Supplementary material 1

Supplementary material 2

Supplementary material 3

Supplementary material 4

Supplementary material 5

Supplementary material 6

Supplementary material 7

Supplementary material 8

Supplementary material 9

Supplementary material 10

Supplementary material 11
