# Supplementary material for: Accelerating bioinformatics implementation in public health
Source: Microb Genom. 2023 Jul 10;9(7):mgen001051. doi: 10.1099/mgen.0.001051 (PMC10438813; doi:10.1099/mgen.0.001051)
Supplement: Supplementary material 11 [file mgen-9-1051-s0011.pdf]

Supplementary Information

Use of demo and validation workspaces

The demo workspace contains the data and analyses undertaken for this manuscript for assessment by readers (https://app.terra.bio/#workspaces/theiagen-demos/MGen-Theiagen-2023). Validation workspaces are produced by Theiagen for every new workflow version release. The validation workspace highlighted in this manuscript (https://app.terra.bio/#workspaces/theiagen-validations/PHBG_Validation_v1-1-00) has been made openly available to demonstrate how users can dependably execute particular versions of workflows on carefully selected datasets.

The demo and validation workspaces described in this manuscript have been made into publicly-available "featured workspaces" so that they can be viewed by anyone and so this can be achieved without logging into Terra. By making these workspaces public, viewers can assess the workspace data, workflow set up and job history. Should you wish to run workflows in either workspace, you will need to create a Terra account and log in, then clone the workspace. You will then have ownership of the cloned workspace and be able to upload data and run workflows in this workspace. Google provides $300 free Google credits that can be used to analyse data in the workspace, though it should be noted that use of the free credits is associated with slow workflow run times. Alternatively, you may set up your own billing project and configure the compute environment to your own needs.

How to execute workflows in Terra

These instructions are very general, for running a workflow in Terra. Slight variations may be required depending on the type of analysis and the resources required. For

additional information regarding the execution of workflows on Terra, please refer to the Terra Support Documentation, https://support.terra.bio/hc/en-us/articles/360036379771-Overview-Running-workflows-in-Terra.

To execute a bioinformatics workflow through the Terra web application

1. Sign in to Terra: Visit the Terra platform at https://app.terra.bio and sign in using your Google account or create a new account if you don't have one.
2. Create or select a workspace: Once signed in, you can create a new workspace by clicking on the "Create a New Workspace" button or select an existing workspace from the list of workspaces you have access to.
3. Upload your sequence data into Terra
    a. Navigate to the "Data" tab: Click on the "Data" tab in the workspace dashboard.
    b. Upload the data: To upload data to the workspace, navigate to the "DATA" tab and click on the "IMPORT DATA" button. You can upload data in several ways:
        i. Upload TSV: For metadata and for sequence data that is already stored in a Google bucket that is available to you. Follow the instructions in the pop-up window.
        ii. Open data uploader: Follow instructions to upload sequence data and a TSV metadata file.
4. Import a workflow: To import a workflow, click on the "Workflows" tab in your workspace. Click on the "Find a Workflow" button to search for existing workflows in the Dockstore or Broad Methods repositories, or import your own WDL file by clicking on the "+" button next to "Create New Workflow."
    a. All workflows mentioned in this manuscript are accessible through the Dockstore Organization page for Theiagen Genomics: https://dockstore.org/organizations/Theiagen
5. Configure the workflow:
    a. Select the version of the workflow you wish to use from the workflows tab of your workspace.

b. Select “Run workflow(s) with inputs defined by data table”.

c. Select from the “Root entity type” dropdown menu the data table where you stored the data that you want the workflow to run on.

d. Click the “SELECT DATA” button, then select the individual samples in the pop-up window.

e. Check the “ Ignore empty outputs” box.

f. Complete the required fields in the INPUTS form.

Note: Determining how to define workflow input and outputs is highly-dependent on the workflow itself. For information about inputs for specific workflows mentioned in this manuscript, please refer to the Theiagen Public Health Resources page (https://theiagen.com/public-health-resources)

g. Select the OUTPUTS form and select “Use defaults”.

h. Select “SAVE”.

i. Select “RUN ANALYSIS”.

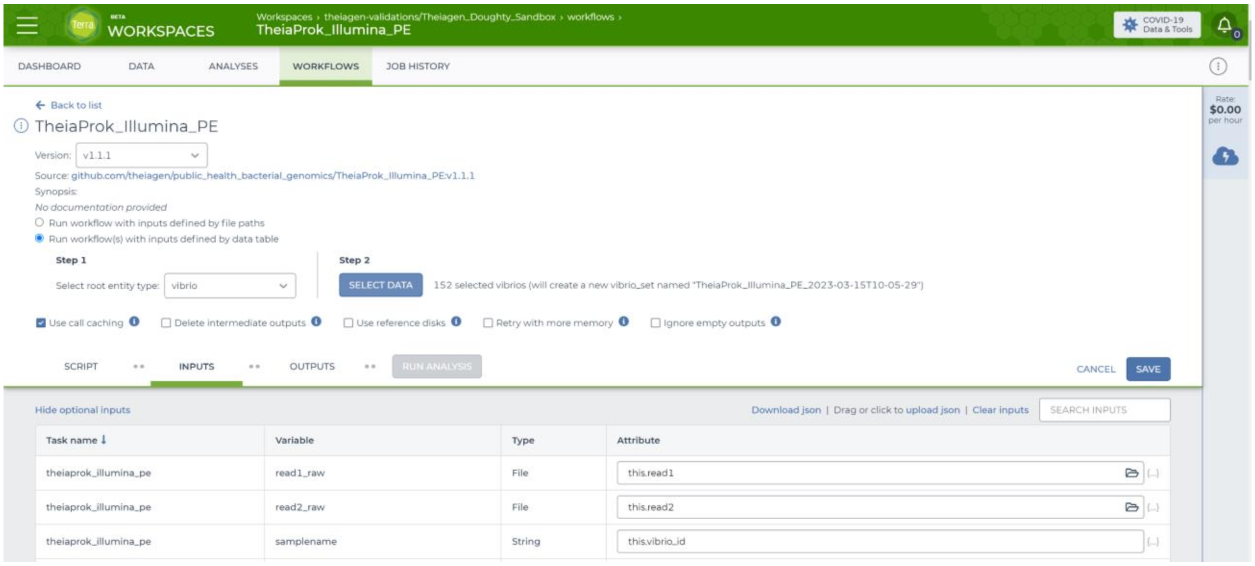

6. Launch the workflow: Click the "Run Analysis" button and in the pop-up, add any notes about your workflow configuration and click the "LAUNCH" button to start the workflow execution.

7. Monitor the progress: After launching the workflow, you can monitor its progress using the "JOB HISTORY" tab in the workflow editor. This tab provides real-time updates on the status of the workflow, including any errors or warnings.

8. View the outputs: After the workflow completes, you can view the output files in the "DATA" tab of the workspace dashboard in the relevant data table.

How to execute Terra workflows through the command-line interface

The workflows mentioned in this manuscript can also be run on the command line using WDL engines such as Cromwell or miniWDL. **Please note that the use of Cromwell cannot be executed behind a proxy. Cromwell requires a direct network connection for proper functionality, and attempting to use it behind a proxy may result in errors or failures.** Detailed documentation on how to install, setup, and use both Cromwell and miniWDL for executing WDL workflows can be found at the public Read The Docs page for these WDL engines: https://miniwdl.readthedocs.io/en/latest/# and https://cromwell.readthedocs.io/en/stable/.

To run the TheiaProk_Illumina_PE workflow using the Cromwell engine, for example, follow these steps:

1. Ensure that Cromwell, Java, and Docker have been installed on your system.
2. Clone the Git repository (this will require you to have previously installed git):
   ```
   git clone
   https://github.com/theiagen/public_health_bacterial_genomics.git
   ```
3. Ensure access to your input files: the TheiaProk_Illumina_PE workflow requires 1) a forward read (read1_raw), 2) a reverse read (read2_raw), and 3) a sample name (samplename). For additional information on optional inputs for the TheiaProk_Illumina_PE workflow, please refer to the technical documentation (https://theiagen.notion.site/TheiaProk-Workflow-Series-623e98b7714f4f07afd70e5086b417e1).

4. Create an input.json file to define these required inputs. For example, to run TheiaProk_Illumina_PE on a pair of read data (sample01_R1.fastq.gz and sample01_R2.fastq.gz) for a hypothetical "sample01", create the following input file:

```
{
    "theiaprok_illumina_pe.samplename": "samplename01",
    "theiaprok_illumina_pe.read1_raw": "path_to_read1",
    "theiaprok_illumina_pe.read2_raw": "path_to_read2"
}
```

5. Use the Cromwell `run` command to execute TheiaProk_Illumina_PE while defining the input.json file, e.g. : `java -jar cromwell-85.jar run public_health_bacterial_genomics/workflows/wf_theiaprok_illumina_pe.wdl -i input.json`
6. Once your workflow has completed, you can find the output files in a workflow subdirectory within the cromwell-executions directory created at workflow execution. This is populated to your current working directory, by default.
